# Schuylkill Valley Veterinary Medical Association

**Published:** 1898-04

**Authors:** I. C. Newhard

**Affiliations:** Acting Secretary


### SCHUYLKILL VALLEY VETERINARY MEDICAL ASSOCIATION.

THE regular quarterly meeting was held on Wednesday, March 16, 1898, at the Commercial Hotel, Shenandoah, Pa. The meeting was called to order by President Noack at 1 P.M. The following members responded to roll-call: McCarthy, Noack, Longacre, Potteiger, Schneider, Newhard. The minutes of the previous meeting were read and approved.

Under admission of new members, the name of Dr. Longacre, of Mantz, Pa., was presented by the Secretary, and his application handed to the Trustees for examination. After recess of a few minutes the applicant was reported favorable, and, after report, duly elected to membership. Dr. W. S. Longacre was also present and was introduced to the members.

The following interesting papers were read: "Meat- and Milk-inspection," by Dr. Noack; "Colic," by Dr. Longacre, followed by the reports of cases: "Chondroma in the Larynx of a Mule," "Fracture of the Patella of a Mule," and "Chronic Valvular Affection of the Heart of a Mule," by Dr. Newhard; "Inversion the Uterus of a Cow," and "Bursitis Intertubercularis," by Dr. Schneider. Papers for the next meeting will be read by Drs. McCarthy, Potteiger, Schneider, and Newhard.

Meeting adjourned at 4.30 P.M., and agreed to meet in Pottsville in June.

I. C. NEWHARD,
Acting Secretary.

